# Soluble epoxide hydrolase gene deletion improves blood flow and reduces infarct size after cerebral ischemia in reproductively senescent female mice

**DOI:** 10.3389/fphar.2014.00290

**Published:** 2015-01-15

**Authors:** Kristen L. Zuloaga, Wenri Zhang, Natalie E. Roese, Nabil J. Alkayed

**Affiliations:** Department of Anesthesiology and Perioperative Medicine, The Knight Cardiovascular Institute, Oregon Health and Science UniversityPortland, OR, USA

**Keywords:** cerebral ischemia, soluble epoxide hydrolase, epoxyeicosatrienoic acids, cerebrovascular, stroke, aging

## Abstract

Soluble epoxide hydrolase (sEH), a key enzyme in the metabolism of vasodilatory epoxyeicosatrienoic acids (EETs), is sexually dimorphic, suppressed by estrogen, and contributes to underlying sex differences in cerebral blood flow and injury after cerebral ischemia. We tested the hypothesis that sEH inhibition or gene deletion in reproductively senescent (RS) female mice would increase cerebral perfusion and decrease infarct size following stroke. RS (15–18 month old) and young (3–4 month old) female sEH knockout (sEHKO) mice and wild type (WT) mice were subjected to 45 min middle cerebral artery occlusion (MCAO) with laser Doppler perfusion monitoring. WT mice were treated with vehicle or a sEH inhibitor t-AUCB at the time of reperfusion and every 24 h thereafter for 3 days. Differences in regional cerebral blood flow were measured *in vivo* using optical microangiography (OMAG). Infarct size was measured 3 days after reperfusion. Infarct size and cerebral perfusion 24 h after MCAO were not altered by age. Both sEH gene deletion and sEH inhibition increased cortical perfusion 24 h after MCAO. Neither sEH gene deletion nor sEH inhibition reduced infarct size in young mice. However, sEH gene deletion, but not sEH inhibition of the hydrolase domain of the enzyme, decreased infarct size in RS mice. Results of these studies show that sEH gene deletion and sEH inhibition enhance cortical perfusion following MCAO and sEH gene deletion reduces damage after ischemia in RS female mice; however this neuroprotection in absent is young mice.

## Introduction

Stroke is recognized as a sexually dimorphic disease, with more women being affected than men (Bushnell et al., 2014). This increased prevalence in women is partially due the fact that women live longer than men. However, women also tend to have poorer outcomes following stroke than men (Reeves et al., 2008). Accordingly, stroke is the 3rd leading cause of death for women, but only the 5th leading cause of death for men (Bushnell et al., 2014). This sex difference has been recognized as a clinically significant issue, as evidenced by the recent publication of the American Heart Association's first ever guidelines for stroke prevention specifically in women (Bushnell et al., 2014). Cerebrovascular events and stroke-related deaths in women increase sharply after menopause (Lisabeth and Bushnell, 2012). Although rodent studies have repeatedly shown that estrogen protects against damage following cerebral ischemia (Liu et al., 2009b), clinical trials failed to show a benefit of hormone replacement therapy in post-menopausal women (Yang et al., 2013). It has been hypothesized that this failure was due to the timing of the hormone replacement (Suzuki et al., 2007), an idea that is supported by recent rodent studies (Liu et al., 2012).

To circumvent the issues related to administering exogenous estrogen, it may be possible to inhibit downstream vascular injury mechanisms that are selectively induced in reproductively senescent (RS) females by loss of estrogen. One potential candidate gene that is regulated by estrogen is soluble epoxide hydrolase (sEH), the enzyme responsible for the breakdown of vasoprotective epoxyeicosatrienoic acids (EETs) into inactive dihydroxyeicosatrienoic acids (DHETs). EETs can protect the brain from ischemic injury by multiple mechanisms including vasodilation, cytoprotection, and suppression of post-ischemic inflammation (Iliff and Alkayed, 2009). Estradiol reduces both basal and post-ischemic sEH expression in females, an effect which may be neuroprotective (Koerner et al., 2008).

sEH has been shown to play a role in stroke risk and ischemic outcome. In humans, sEH gene (EPHX2) polymorphisms have been shown to alter stroke incidence (Gschwendtner et al., 2008; Fava et al., 2010) and ischemic outcome (Koerner et al., 2007). Further, sEH inhibition has been shown to reduce infarct size in young male stroke-prone spontaneously hypertensive rats (Dorrance et al., 2005; Simpkins et al., 2009) and young male mice (Zhang et al., 2007, 2009; Jouihan et al., 2013). In addition, in young male mice sEH gene deletion also reduces infarct size (Zhang et al., 2008). Conversely, these protective effects of sEH gene deletion are not observed in young intact female mice but are observed in ovariectomized female mice (Zhang et al., 2009), suggesting that ovarian hormones may provide protection by reducing sEH expression.

Since estradiol levels decline with age (Barron and Pike, 2012; Lisabeth and Bushnell, 2012), we hypothesized that sEH inhibition/gene deletion may be protective in RS female mice similar to what we have observed in ovariectomized mice. We hypothesized that loss of estrogen after RS would remove the inhibitory effect of estrogen on sEH and increase its expression. We further hypothesized that sEH inhibition or gene deletion in RS female mice would increase cerebral perfusion and decrease infarct size following stroke. Our data show that although neither estrogen levels nor sEH expression changed with age in our mouse model, sEH gene deletion enhances cerebral perfusion in RS females and protects against ischemic damage.

## Materials and methods

This study was conducted in accordance with the National Institutes of Health guidelines for the care and use of animals in research, and protocols were approved by the Institutional Animal Care and Use Committee at Oregon Health and Science University, Portland, OR, USA.

### Animal model

Young (3–4 month old), middle aged (11–12 month old), and RS (18–22 month old) female C57BL/6 (wild type; WT) mice purchased from Charles River were used for sEH protein analysis studies. For stroke studies, WT mice were purchased as C57BL/6 mice from Charles River and as C57BL/6J mice from Jackson laboratories depending on the comparison group. For aging studies not involving transgenic mice, the C56BL/6 mice were used. For aging studies involving sEHKO mice, which are on a C57BL/6J background, C56BL/6J mice were used. Age-matched RS (15–18 month old) or young (3–4 month old) female mice were used. The sEH inhibitor trans-4-[4-(3-Adamantan-1-yl-ureido)-cyclohexyloxy]-benzoic acid (t-AUCB) was developed and generously provided by Dr. Bruce Hammock, University of California, Davis, CA (Liu et al., 2009a). Vehicle (4% PEG400 in water) or t-AUCB (1 mg/kg) were administered i.p. at the time of reperfusion and every 24 h thereafter for 3 days. Homozygous sEHKO mice were generated from an in-house breeding colony. Genotype was confirmed by PCR, as previously described (Sinal et al., 2000).

### Transient middle cerebral artery occlusion

Transient focal ischemia was achieved by occluding the middle cerebral artery for 45 min as previously described (Zhang et al., 2009). Briefly, mice were anesthetized with isoflurane and kept warm with water pads. Body temperature was maintained at 37 ± 0.5°C. A small laser-Doppler probe was affixed to the skull to monitor cortical perfusion and verify vascular occlusion and reperfusion. Unilateral middle cerebral artery occlusion (MCAO) was achieved by advancing a 6-0 nylon monofilament suture into the right internal carotid artery via the external carotid artery stump until the laser-Doppler signal dropped to <20% of baseline.

### Optical microangiography

OMAG was performed 24 h prior to ischemia and again 24 h after MCAO as previously described (Iliff et al., 2009; Zhang et al., 2009). Blood perfusion was visualized and quantified based on endogenous light scattering from moving blood cells within the brain. We used a superluminescent diode with a central wavelength of 1, 310 nm and a full-width, half-maximum bandwidth of 50 nm to illuminate the OMAG system. The spectral interferograms formed by the reference light and the light backscattered from the tissue sample were detected by a custom-built, high-resolution and high-speed spectrometer. A final volume data cube of 1000 × 500 × 512 (*x* − *y* − *z*) voxels was built from which the three-dimensional OMAG structural and flow images were computed. The three-dimensional scan represented a physical volume with the following *x* − *y* − *z* dimensions: 2.5 × 2.5 × 2.0 mm^3^.

### Measurement of infarct size

Infarct size was measured by 2,3,5-triphenyltetrazolium chloride (TTC) stain 72 h after reperfusion, as previously described (Zhang et al., 2009). To estimate infarct size, olfactory bulbs and cerebellum were removed and the brain was sectioned into four equally spaced 2-mm coronal sections (between +3 and −5 mm relative to bregma). TTC stained brain sections were photographed, and infarcted (unstained) areas were measured with MCID (InterFocus Imaging Ltd, Linton, Cambridge, UK) imaging software. To account for the effect of edema, we estimated infarct size indirectly by subtracting the uninfarcted area in the ipsilateral hemisphere from the total area of the contralateral hemisphere.

### Hormone assays

Serum 17-beta estradiol (E2) levels and testosterone (T) levels were measured at the Endocrine Technology Support Core Lab at the Oregon National Primate Research Center at OHSU. Extraction-radioimmunoassay (Ext-RIA) with liquid chromatographic separation of different steroids before the Ext-RIA was used (Rasmussen et al., 1984). This technique is sample volume-independent; where higher steroid levels in the sample can be detected with less volume. Briefly, mouse serum or plasma samples (20 to 200 μl) were brought to 300 μl with 0.1% bacto-gelatin in phosphate buffered saline (PBS) and then 5 ml redistilled diethyl ether was added to each sample. After shaking for 3 min, samples were centrifuged and then frozen in a dry ice-ethanol bath. The ether-soluble fraction was decanted from the frozen aqueous fraction into a collecting tube, and dried under forced air. The extract was then re-dissolved in 200 μl of column solvent (Hexane:benzene:methanol = 62:20:13) and added to a 1 × 6 cm all-glass column containing 1 g Sephadex LH-20 for separation of steroids. Each fraction was dried under forced air stream and subjected to its respective RIA, i.e., an ultrasensitive E2 RIA with sensitivity at 1 pg/tube or a T RIA with sensitivity at 3 pg/tube. Hormonal values, expressed as pg/ml for E2 or ng/ml for T, were corrected for extraction-chromatography losses determined by radioactive trace recovery performed simultaneously with sample extraction; hot recovery is usually between 60 and 80%. The overall inter-assay variation for steroid extraction radioimmunoassays was less than 15% and the intra-assay variations did not exceed 10%.

### Western blot

Mice were perfused with ice-cold heparinized saline to remove blood from brains prior to tissue collection. Brains were homogenized in lysis buffer containing sucrose (250 mM), potassium chloride (60 mM), tris-hydrochloride (15 mM), sodium chloride (15 mM), ethylenediaminetetraacetate (EDTA, 5 mM), ethylene glycol tetraacetic acid (EGTA, 1 mM), phenylmethanesulfonylfluoride (PMSF, 0.5 mM), dithiothreitol (DTT, 10 mM), 1 Complete Mini-EDTA free Protease Inhibitor Cocktail tablet (Roche Diagnostics, Indianapolis, IN), and 10 μl/ml each of phosphatase inhibitor solution 1 and phosphatase inhibitor solution 2 (Sigma-Aldrich, St. Louis, MO). Lysates were then centrifuged at 2000 × g for 10 min at 4°C, supernatant collected and centrifuged at 17,000 × g for 20 min at 4°C, and the remaining supernatant was collected. Protein samples (40 μg) were separated by gel electrophoresis and then transferred to Polyvinylidene Difluoride (PVDF) membranes. Blots were blocked in 5% dry milk, and incubated at 4°C overnight with a primary rabbit polyclonal antibody against murine sEH (1:500; Cayman Chemical, Ann Arbor, MI) or beta actin (1:2000; Sigma-Aldrich). The signal was visualized using a horseradish peroxidase-linked (HRP) secondary antibodies against rabbit (1:1000; GE Healthcare, Salt Lake City, UT), or mouse (1:1000; GE Healthcare) followed by detection using Supersignal chemiluminescent reagents (Thermo Fisher Scientific) with a FluorChem FC2 (Protein Simple, Santa Clara, CA). Blots were stripped using Restore Western Blot Stripping Buffer (Thermo Fisher Scientific) and re-blocked in milk after imaging each blot for sEH and prior to incubation in a subsequent primary antibody for beta actin. Densitometry was quantified with AlphaView software (Protein Simple). Data were normalized to the loading control beta actin, then they were expressed relative to levels in young females.

### Statistical analysis

Data were analyzed using two-way analysis of variance with *post-hoc* Newman–Keuls test for multiple comparisons. The criterion for statistical significance was set at *P* < 0.05. All values are reported as mean ± standard error.

## Results

### sEH expression did not change with age

**Figure 1:**
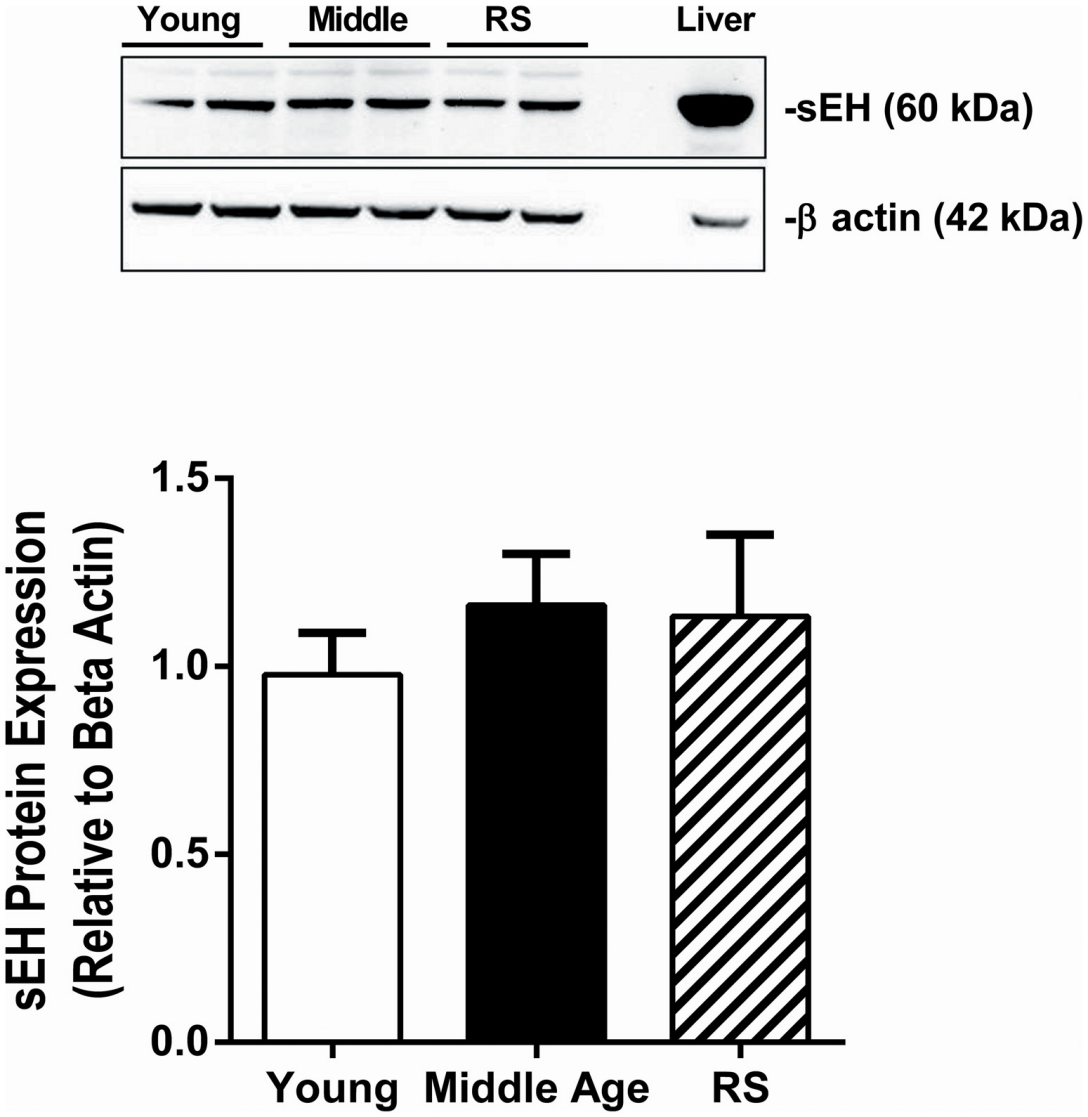
**sEH expression did not change with age**. sEH expression was measured via Western blot in brains collected from young (3–4 months old), middle age (11–12 months old), and reproductively senescent (RS; 18–22 months old) female wild type mice. sEH expression was normalized to beta actin expression and is shown as a ratio relative to expression in young female mice. No significant differences were observed between age groups. *N* = 7–8 per group.

In order to determine if sEH expression in brain tissue changes with age, brains were collected from young (3–4 months old), middle age (11–12 months), and RS (18–22 months old) WT female mice. sEH protein expression in brain, measured via western blot, did not vary with age in female mice (Figure 1).

### Perfusion during MCAO is not altered by sEH inhibition/gene deletion or age

Young (3–4 months old) and RS (15–18 months old) WT and sEHKO mice were subjected to a 45 min MCAO followed by reperfusion. A separate cohort of WT mice received vehicle or the sEH inhibitor t-AUCB (1 mg/kg, i.p.) at the time of reperfusion and every 24 h thereafter for 3 days. Characteristics of study mice including age and body weight are presented in Table 1. Body weight increased with age for all treatment groups. In addition, RS sEHKO mice had elevated body weight compared to WT RS mice. Relative perfusion of the MCA territory was measured by laser Doppler probe continuously at baseline, during the occlusion, and during 5 min post-reperfusion (averages of 5–15 min intervals are shown). Relative perfusion during occlusion and reperfusion was not altered by age, sEH gene deletion, or sEH inhibition (Figure 2).

**Table 1:** **Characteristics of MCAO Study Mice**.

	**Age (months)**	**Body weight (g)**
WT young	3–4	19.6 ± 0.4
sEHKO young	3–4	20.4 ± 0.3
WT RS	17–18	25.5 ± 0.5^*^
sEHKO RS	15	29.2 ± 1.0^*^^#^
Veh young	3–4	21 ± 0.4
t-AUCB young	3–4	20.9 ± 0.3
Veh RS	17–18	29.4 ± 0.7^*^
t-AUCB RS	17–18	29.2 ± 1.1^*^

*Age range and average body weight, during MCAO are shown for each experimental group. WT, wild type; sEHKO, soluble epoxide hydrolase knockout; RS, reproductively senescent*.

* *p < 0.05 vs. young mice of same treatment group*.

# *p < 0.05 compared to WT RS mice*.

**Figure 2:**
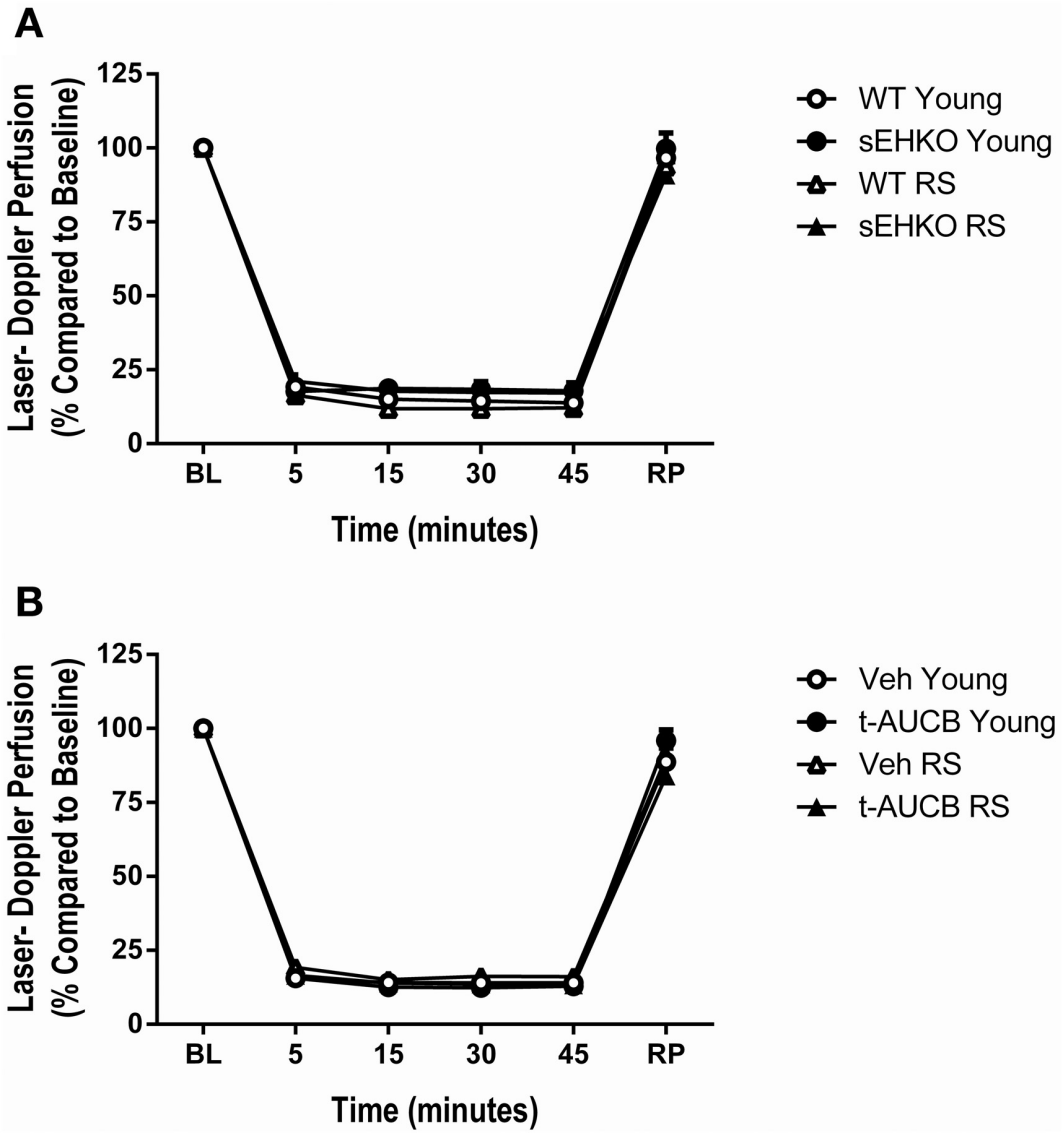
**Perfusion during MCAO is not altered by sEH inhibition/gene deletion or age**. Relative perfusion, measured by laser Doppler probe, of the MCA territory measured continuously (averages of 5–15 min intervals are shown) at baseline, during the occlusion, and during 5 min post-reperfusion in young and reproductively senescent (RS) WT and sEHKO mice **(A)** and WT mice treated with vehicle or the sEH inhibitor t-AUCB **(B)**. **(A)** Perfusion during occlusion and reperfusion was not altered by age or sEH gene deletion. **(B)** Perfusion during occlusion and reperfusion was not altered by age or sEH inhibition. *N* = 9–15 per group.

### sEH inhibition/gene deletion increases cortical perfusion 24 h after MCAO

**Figure 3:**
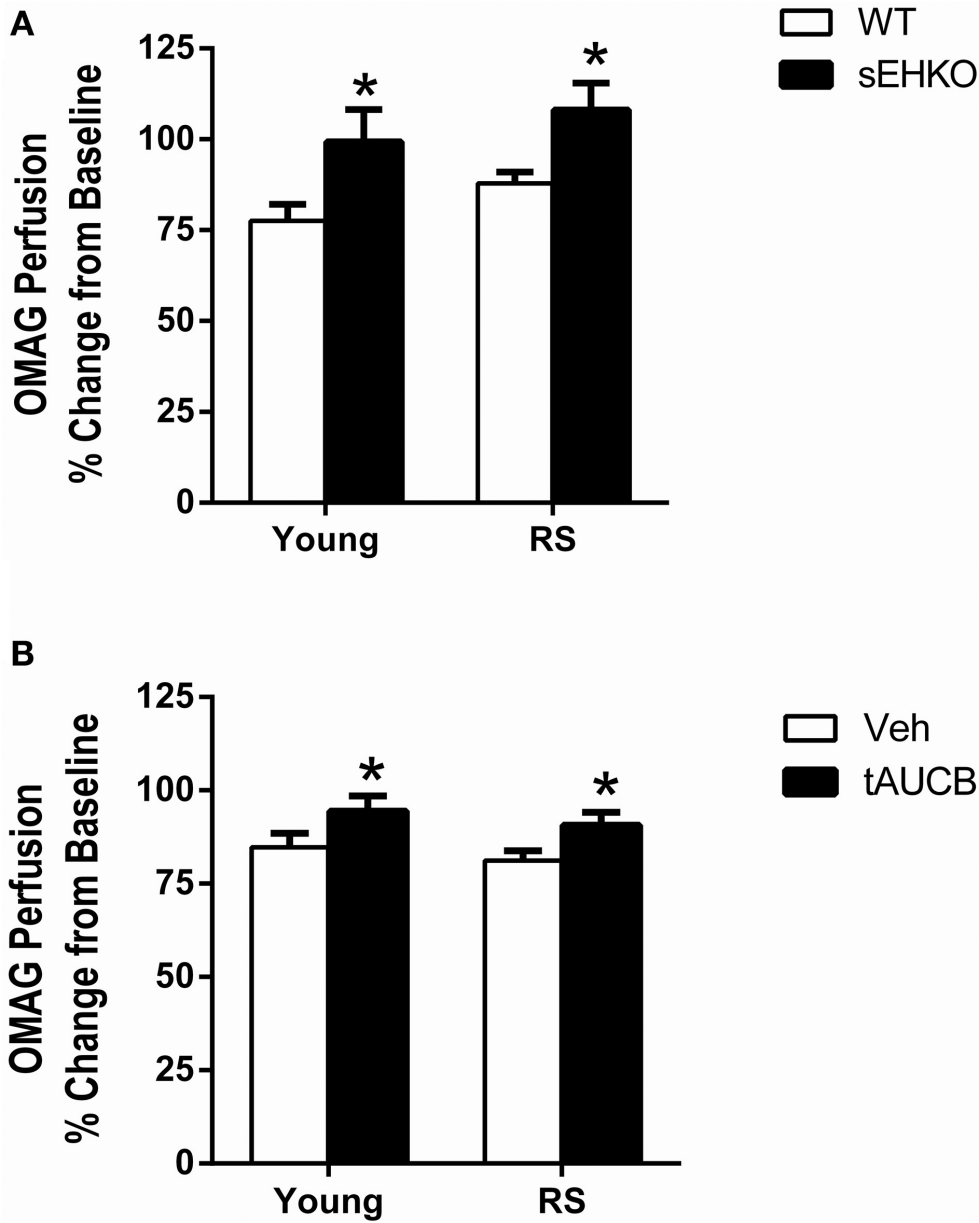
**sEH inhibition/gene deletion increases cortical perfusion 24 h after MCAO**. Cortical perfusion was measured via optical microangiography (OMAG) in young and reproductively senescent (RS) WT and sEHKO mice **(A)** and WT mice treated with vehicle or the sEH inhibitor t-AUCB **(B)** 24 h prior to MCAO and then again 24 h after MCAO. Perfusion is shown as a percent change from the baseline scan. **(A)** Post-ischemic cortical perfusion was not altered by age in WT mice. Post-ischemic cortical perfusion was increased in sEHKO mice compared to WT mice of both age groups (^*^p < 0.01). **(B)** Post-ischemic cortical perfusion was not altered by age in vehicle-treated WT mice. Post-ischemic cortical perfusion was increased by sEH inhibition in WT mice (^*^p < 0.05). *N* = 8–12 per group.

We have previously shown that cortical perfusion is higher in young females compared to young males after MCAO and that this effect is mediated by sEH in male mice (Zhang et al., 2009). We sought to determine if sEH also played a role in post-ischemic cortical perfusion in aged female mice. 24 h after MCAO, cortical perfusion was measured via optical microangiography (OMAG) in young and RS WT and sEHKO mice and WT mice treated with vehicle or the sEH inhibitor t-AUCB at the time of reperfusion and then treated again 24 h later. Cortical perfusion was not altered by age. However, cortical perfusion was increased in sEHKO mice compared to WT mice [Figure 3A, *F*_(1, 37)_ = 9.38, *p* < 0.01]. Cortical perfusion was also enhanced in WT mice that received treatment with the sEH inhibitor compared to vehicle treated WT mice [Figure 3B, *F*_(1, 36)_ = 6.86, *p* < 0.05].

### sEH gene deletion reduces infarct size 72 h after MCAO in RS female mice

**Figure 4:**
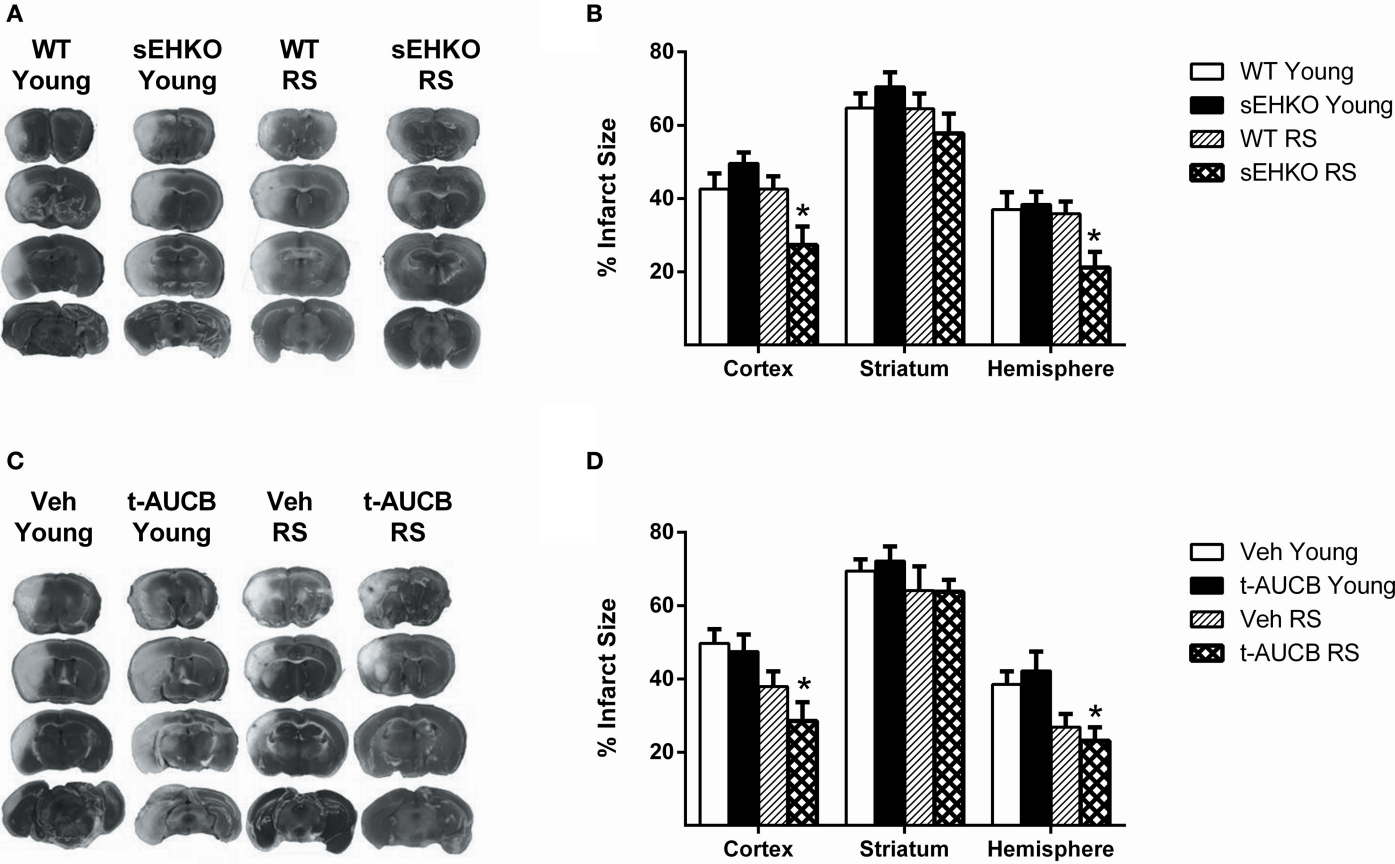
**sEH gene deletion reduces infarct size 72 h after MCAO in RS female mice**. Three days after MCAO, infarct size was measured via TTC stain in young and reproductively senescent (RS) WT and sEHKO mice **(A,B)** and WT mice treated with vehicle or the sEH inhibitor t-AUCB **(C,D)**. **(A)** Representative infarct sizes in WT and sEHKO mice. **(B)** Infarct size was not altered by age in WT mice or by sEH gene deletion in young mice. In RS mice, infarct size was decreased in the cortex and total hemisphere of sEHKO mice compared to WT mice (^*^p < 0.05). **(C)** Representative infarct sizes in vehicle and t-AUCB treated mice. **(D)** Infarct size was not altered by age in vehicle-treated WT mice or by sEH inhibition in young mice. In RS mice, sEH inhibition decreased infarct size in both the cortex and total hemisphere compared to young vehicle-treated mice, but not compared to RS vehicle-treated mice (^*^p < 0.05). *N* = 9–15 per group.

We have previously shown that in young intact female mice sEH gene deletion does not alter infarct size after MCAO, while in ovariectomized female mice sEH gene deletion did decrease infarct size (Zhang et al., 2009). However, the effects of sEH gene deletion/inhibition in aged females are unknown. We compared infarct size 72 h after MCAO in young and RS female WT and sEHKO mice. A main effect of genotype was detected [*F*_(3, 114)_ = 9.99, *p* < 0.0001]. *Post-hoc* test revealed, as previously observed, that sEH gene deletion had no effect on infarct size in young intact mice (Figures 4A,B). In addition, infarct size was not altered by age in WT mice. However, RS sEHKO mice had significantly decreased infarct size in both the cortex and the total hemisphere compared to RS WT mice and young WT mice (Figures 4A,B, *p* < 0.05). Next, we determined if sEH inhibition, when administered after the ischemic event, could provide protection in aged female mice. The sEH inhibitor t-AUCB (1 mg/kg; i.p.) or vehicle treatment was administered at the time of reperfusion and then every 24 h thereafter for 3 days prior to infarct analysis. A main effect of treatment was detected [*F*_(3, 135)_ = 9.36, *p* < 0.0001]. *Post-hoc* tests revealed that, similar to sEH gene deletion, sEH inhibition also did not provide protection in young female mice. However, in RS mice sEH inhibition reduced infarct size in both the cortex and the total hemisphere compared to young mice with vehicle treatment but not in comparison to RS mice treated with vehicle (Figures 4C,D, *p* < 0.01).

### Effects of sEH inhibition/gene deletion on serum hormone levels 72 h after MCAO

**Figure 5:**
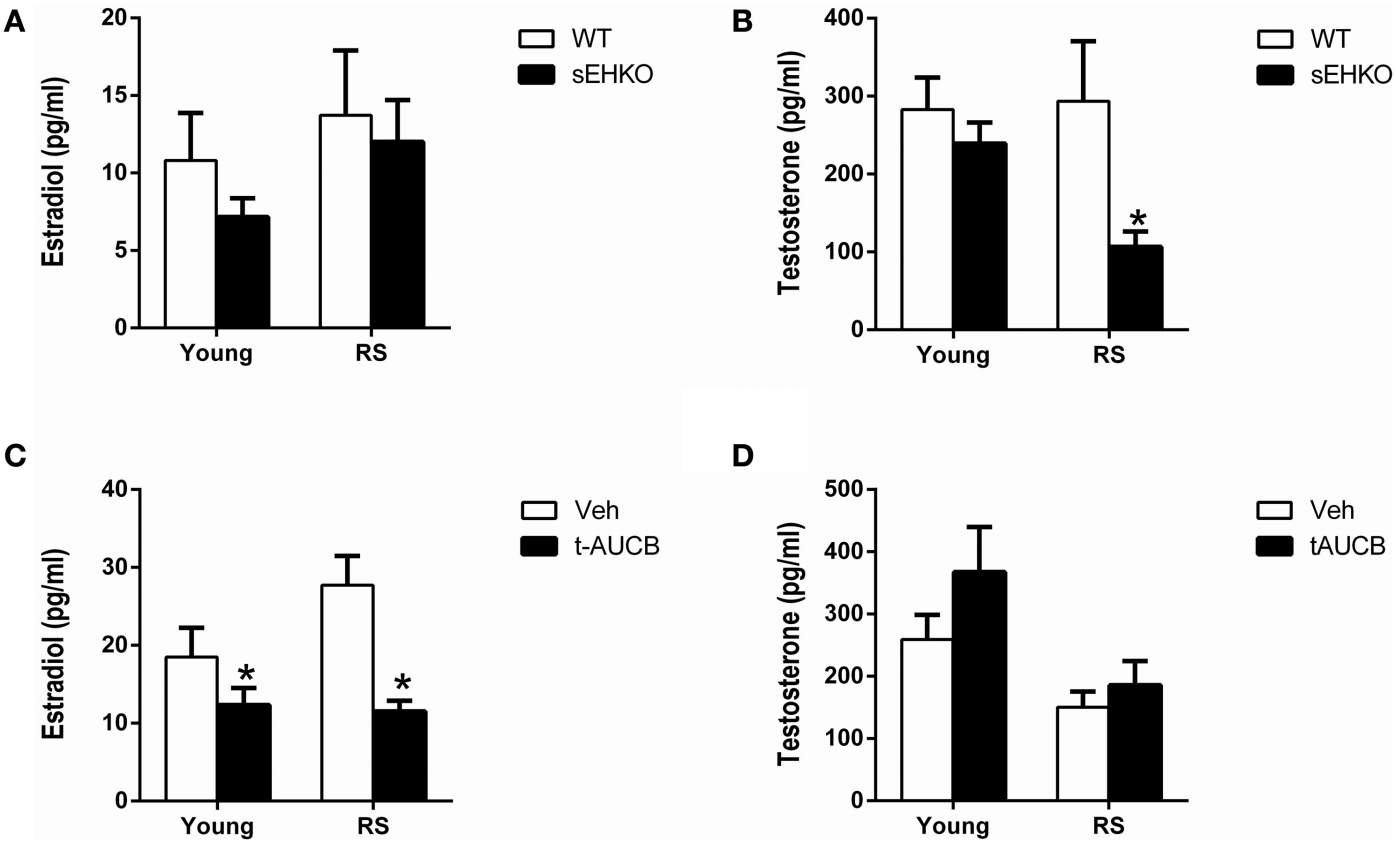
**Effects of sEH inhibition/gene deletion on serum hormone levels 72 h after MCAO**. Three days after MCAO, serum estradiol **(A,C)** and testosterone **(B,D)** levels were measured in young and reproductively senescent (RS) WT and sEHKO mice **(A,B)** and WT mice treated with vehicle or the sEH inhibitor t-AUCB **(C,D)**. **(A)** Estradiol levels were not altered by age or sEH gene deletion. **(B)** Testosterone levels were not altered by age or by sEH gene deletion in young mice. In RS mice, sEHKO mice had decreased testosterone levels compared to WT mice (^*^p < 0.05). **(C)** Estradiol levels were decreased by sEH inhibition in both young and RS mice (^*^p < 0.05). **(D)** Testosterone levels were not altered by age or sEH inhibition in WT mice. *N* = 10–15 per group.

Since estradiol is known to provide protection from ischemic damage in young female mice, if sEH inhibition or gene deletion significantly altered estradiol levels this could potentially have contributed to the observed differences in infarct size between groups. Serum estradiol and testosterone levels were measured 72 h after MCAO in young and RS WT and sEHKO female mice and in young and RS WT female mice treated with vehicle or the sEH inhibitor t-AUCB. Estradiol levels were not altered by age or sEH gene deletion (Figure 5A). However, a main effect of genotype was found for testosterone levels [*F*_(1, 38)_ = 5.68, *p* < 0.05], with *post-hoc* test revealing significantly decreased testosterone in RS but not young sEHKO mice compared to WT mice (Figure 5B, *p* < 0.05). A main effect of t-AUCB treatment on estradiol levels was detected [*F*_(1, 46)_ = 13.13, *p* < 0.001], with *post-hoc* tests revealing that estradiol levels were significantly decreased by sEH inhibition compared to vehicle in both young and RS mice (Figure 5C, *p* < 0.001). Testosterone levels were not altered by sEH inhibition (Figure 5D).

## Discussion

The goal of the current study was to determine if sEH inhibition or gene deletion could improve cerebral perfusion and reduce ischemic damage in aged female mice. We found that sEH gene deletion/inhibition enhances cortical blood flow in both young and RS female mice following cerebral ischemia. Additionally, in RS, but not young mice, sEH gene deletion also reduced infarct size in both the cortex and total hemisphere. These findings suggest that while sEH plays a role in cerebral blood flow response to ischemia in both young and RS females, it does not influence ischemic damage in young females, but provides protection in RS females. Therefore, our current data indicate that the role of sEH in ischemic damage in females is age-dependent.

EETs are known to provide protection against ischemic damage via a variety of neuroprotective mechanisms including: enhanced vasodilation (Iliff et al., 2010), improving cerebrovascular structure and microvascular density (Simpkins et al., 2009), inhibition of platelet adhesion (Heizer et al., 1991), reduction of inflammation (Spector and Norris, 2007), reduction of oxidative stress (Yang et al., 2001), reduction of apoptosis, or activation of protective signal transduction (Iliff et al., 2010; Merkel et al., 2010).

In line with this, sEH gene polymorphisms in the human sEH gene (EPHX2) have been shown to alter stroke incidence (Gschwendtner et al., 2008; Fava et al., 2010) and ischemic outcome (Koerner et al., 2007). Further, we have previously shown that a human variant of the sEH gene that increases sEH enzymatic activity is associated with increased cell death in cortical neurons exposed to oxygen-glucose deprivation and reoxygenation, while a mutation that reduces sEH activity provides protection from neuronal cell death (Koerner et al., 2007). Inhibition of sEH or sEH gene deletion has also been shown to be protective in rodent models of cerebral ischemia. Specifically, sEH inhibition has been shown to reduce infarct size in young male rats (Dorrance et al., 2005; Simpkins et al., 2009) and young male mice when administered as a pre-treatment (Zhang et al., 2007, 2009; Jouihan et al., 2013) or at the time of reperfusion (Zhang et al., 2007). We have shown in young male mice that sEH gene deletion also reduces infarct size (Zhang et al., 2008). sEH inhibition has also been shown to reduce neurological deficits after MCAO in male rats (Simpkins et al., 2009).

sEH appears to contribute to stroke risk and ischemic damage, in part, via a vascular mechanism. We have shown that male sEH knockout (sEHKO) mice have enhanced regional cerebral blood flow during MCAO compared to WT male mice (Zhang et al., 2008). The effects of EETs on ischemic injury also appear to be sexually dimorphic. We have previously shown that cerebrovascular expression and activity of sEH are lower in female than male mice (Zhang et al., 2009). While sEH gene deletion reduces infarct size in young male mice, these protective effects of sEH gene deletion are not observed in young intact female mice but are observed in ovariectomized female mice (Zhang et al., 2009). This lack of protection is young intact females is likely due to the fact that these mice already have very low sEH expression (Zhang et al., 2009) because estrogen suppresses sEH expression (Koerner et al., 2008). We have also shown that cerebral blood flow during MCAO is higher in intact WT female mice compared with male mice and ovariectomized female mice. Sex differences in cerebral blood flow during MCAO were absent in sEHKO mice, due to increased blood flow in the male and ovariectomized female mice (Zhang et al., 2009). This data suggests that ovarian hormones may improve cerebral blood flow via decreases in sEH since ovariectomy does not impair cerebral blood flow in sEHKO mice (Zhang et al., 2009).

Our data show that both sEH gene deletion and sEH inhibition increase post-ischemic cortical perfusion in both young and RS female mice. We have previously shown that sEH gene deletion drastically decreases cerebrovascular hydrolase activity in both male and female mice (Zhang et al., 2009). Furthermore, we have also shown that *in vivo* sEH inhibition with t-AUCB increases EETs concentrations in mouse brain (Jouihan et al., 2013). Since EETs are known to cause vasodilation (Iliff et al., 2010), enhanced vasodilation could be responsible for the observed increase in cortical perfusion. We have also previously shown that sEH gene deletion increases cortical blood flow in young male mice during ischemia but not in young female mice (Zhang et al., 2009). In line this, in the current study we did not detect differences in perfusion with sEH gene deletion or inhibition in female mice in either age group during MCAO or immediately (5 min) after reperfusion. However, differences between groups emerged when cortical perfusion was measured via OMAG 24 h after ischemia. At the 24 h time point, both sEH inhibition and sEH gene deletion increased perfusion in both age groups. Thus, the time course of modulation of cerebral perfusion by sEH following ischemia appears to be sexually dimorphic, emerging early in males, but with delayed onset in females.

In RS female mice sEH gene deletion, but not sEH inhibition, provided protection against ischemic damage. There was no effect for t-AUCB in any age group, although there was a trend toward a decrease in infarct size in tAUCB-treated compared to vehicle RS mice. Taken together, the data in Figures 4A,B suggest that sEH gene deletion, but not pharmacological inhibition, decreases infarct size in RS females. Pharmacological inhibition seems, however, to potentiate the age-related decrease in infarct size observed in C56BL/6 (Figure 4B). A possible explanation for the discrepancy between the effects of sEH gene deletion compared to pharmacological inhibition may be related to the recent discovery that sEH is a bifunctional enzyme containing both phosphatase and hydrolase enzymatic activities (Cronin et al., 2003; Newman et al., 2003). While both of these enzyme activities are abolished by gene deletion, only the hydrolase activity is inhibited by the pharmacological blockers. The other explanation is that life-long deletion of sEH in sEHKO mice may lead to compensatory changes; for example, vascular remodeling, which are not observed following acute pharmacological inhibition (Koerner et al., 2008).

In contrast to the protection observed in RS mice with sEH gene deletion, no effect of was seen in young mice. Thus, the effects of sEH gene deletion appear to be age-dependent in females. The lack of protection in young female mice supports our previous study in which we did not observe differences in infarct size between young female WT and sEHKO mice (Zhang et al., 2009). The lack of reduction infarct size in young mice despite an increase in post-ischemic perfusion seems contradictory. However, there are several potential explanations for this finding. First, although blood flow does play a role in ischemic outcome, there are many other factors that also contribute to infarct size such as inflammation, oxidative stress, and hormonal status. If sEH gene deletion/inhibition did not positively influence these other factors in the young mice, this would explain why the improved blood flow did not lead to neuroprotection. EETs are known to have anti-inflammatory (Spector and Norris, 2007), anti-oxidant (Yang et al., 2001), and anti-apoptotic effects (Iliff et al., 2010; Merkel et al., 2010); therefore, it is possible that sEH gene deletion caused greater neuronal protection through one of these mechanisms in the aged female mice compared to the young female mice. Second, endogenous vascular sEH expression may be lower in young mice than in RS mice. We have previously observed that sEH expression is increased in brains of aged female rats compared to young female rats (unpublished observation). Although, we did not detect significant differences in whole brain sEH expression with age in the current study, there may have been differences in cerebrovascular sEH expression that were diluted by our use of the whole brain instead of isolated vessels. Furthermore, since EETs levels are regulated by both synthesis and degradation, it is possible that although sEH protein expression was unchanged with age EETs levels could have been higher in the young mice. If EETs levels were already high in young mice but not in the RS mice, then sEH inhibition/gene deletion would be expected to have less of an effect in young compared to RS mice. A third possible explanation is that the lower testosterone levels observed with sEH gene deletion in RS mice contributed to the reduction in infarct size, since testosterone has been shown to increase infarct size by increasing glutamate toxicity, inflammation, and apoptosis (Hawk et al., 1998; Yang et al., 2002; Cheng et al., 2007). Reduced estradiol levels observed with sEH inhibition in young mice may have enhanced injury in young mice and masked any protective effects of sEH inhibition, since estradiol is known to protect against ischemic injury through mechanisms including reducing inflammation, oxidative stress, and enhancing neurogenesis and cerebral blood flow in young mice (Alkayed et al., 1998; McCullough et al., 2001; Wen et al., 2004; Liu et al., 2009b; Li et al., 2011). Conversely, reduced estradiol levels may not enhance injury in RS mice, since the protective effects of estradiol appear to decrease with age (Selvamani and Sohrabji, 2010; Liu et al., 2012).

Few studies have compared stroke outcome in young and RS female rodents. We did not observe an age-related change in ischemic sensitivity. Our results are in line with previous reports that showed no differences in infarct size between young and RS females (Dubal and Wise, 2001; Manwani et al., 2013). However, other studies have shown that aged female mice tend to have larger infarct size than young female mice (Selvamani and Sohrabji, 2010; Selvamani et al., 2014). Many of these studies have been conducted in middle aged mice (9–12 months old) that are younger than the 15–18 months old female mice used in the current study. Estradiol levels are known to decrease with age (Barron and Pike, 2012; Lisabeth and Bushnell, 2012). We did not detect an age-related decline in estradiol levels in the current study. It has been reported that the transition to reproductive senescence is associated with loss of cyclicity, rather than decrease in peak hormone levels (Wise et al., 1994). Loss of cyclicity could not have been detected in our studies, which used a single terminal measurement of hormones. Furthermore, at the time of blood collection, the young female mice displayed hormone levels consistent with diestrus, the phase of their estrus cycles in which estradiol levels are lowest (Walmer et al., 1992), or RS. It has been demonstrated that stress increases the time spent in diestrus in mice (Breen et al., 2012). In addition, it is well-documented that stress decreases estradiol production in both humans and rodents (Whirledge and Cidlowski, 2010). Therefore, when serum was collected 72 h after the stroke surgery estradiol levels may have been very low due to severe stress inhibiting the hypothalamic-pituitary-gonadal axis. Since estradiol levels are already very low in RS mice, we would not expect to see as much of a decrease in estradiol levels in RS mice after stress. We also did not detect age-related changes in testosterone levels. However, sEH inhibition, but not gene deletion, did reduce estradiol levels in both young and RS mice. Whether or not this reduction in estradiol level contributed to infarct size is unknown. In addition, sEH gene deletion also reduced testosterone levels in RS, but not young, mice. As previously mentioned, since testosterone has been reported to increase infarct size (Hawk et al., 1998; Yang et al., 2002; Cheng et al., 2007), this reduction in testosterone levels could potentially have contributed to the reduction in infarct size observed in RS mice with sEH gene deletion.

In summary, we have shown that sEH gene deletion enhances cortical blood flow and reduces infarct size in RS female mice following cerebral ischemia. Further, we have shown that although sEH inhibition/gene deletion also increases cortical blood flow in young female mice, this effect does not elicit neuroprotection. Therefore, our current data indicate that the role of sEH in protecting against ischemic damage in females is age-dependent. Understanding the downstream injury mechanisms elicited with age in females could lead to new therapeutic strategies for stroke to improve cerebral blood flow and reduce ischemic damage in post-menopausal women.

### Conflict of interest statement

The Review Editor Matthew L. Edin declares that, despite having collaborated with author Nabil J. Alkayed, the review process was handled objectively and no conflict of interest exists. The authors declare that the research was conducted in the absence of any commercial or financial relationships that could be construed as a potential conflict of interest.
